# Radiation Therapy and its Effects Beyond the Primary Target: An Abscopal Effect

**DOI:** 10.7759/cureus.4100

**Published:** 2019-02-19

**Authors:** Shaqul Qamar Wani, Ishtiyaq A Dar, Talib Khan, Mohammad M Lone, Fir Afroz

**Affiliations:** 1 Radiation Oncology, Sher I Kashmir Institute of Medical Sciences, Srinagar, IND; 2 Anesthesiology, Sher I Kashmir Institute of Medical Sciences, Srinagar, IND

**Keywords:** radiation therapy, abscopal effect, immunomodulation, curative intent, metastatic disease, immunotherapy

## Abstract

Radiation therapy (RT) has been used for the treatment of various malignancies since decades with curative or palliative intent. RT for primary disease is often used with curative intent while its use in metastatic settings has been essentially palliative. However, in certain malignancies with metastatic disease, RT to primary disease has led to the regression of not only the primary site but also of the metastatic sites, a phenomenon known as “abscopal effect.” Keeping in view the positive effects of RT beyond the primary site, we review the clinical utility of RT regarding its abscopal effect.

## Introduction and background

The abscopal effect refers to the regression of other tumor foci that were outside the initial localized radiation treatment field and a phenomenon originally described by the British radiologist R.H. Mole in 1953. He observed that cell death occurs in two ways, when mammals were subjected to irradiation. The long-understood method of delayed cell death due to radiation interference with cell division was no new discovery, but he also observed early death in cells that normally did not divide [1]. The presumed mechanism of the abscopal effect has long been thought to be immune related, but definitive proof of this theory was uncertain. Discoveries in the recent past have supported the propositions that this effect is primarily immune mediated. Patients with several distinct cancer histologies and across a range of ages have benefited from this phenomenon. The abscopal response is now being probed actively with an objective to improve the therapeutic outcomes of metastatic cancers, especially in combination with emerging immunotherapy (IT) agents [2].

## Review

Hypothesis and mechanism of abscopal effect and immunomodulation

An understanding of the basic principles of radiotherapeutic effectiveness is essential in comprehending its role in the abscopal effect [3]. The basic intention of RT is to deliver calculated quantum of radiation dose that is tumoricidal coupled with limiting damage to the surrounding normal tissues. A conventional fractionation, with daily doses of 1.8 to 2 Gy is often used. Radiation energy is absorbed and causes affected electrons to be raised to a higher energy state within the atoms within a tumor. Ionization causes cell kill either by a) mitotic cell death caused by generation of free radicals leading to DNA damage, b) bystander effect causing cellular damage transmitted to a adjacent cell via communicating gap junctions [4-7], or c) radiation-induced vascular fibrosis and occlusion [8-9]. Evidence suggests that the primary mechanism driving the loss of tumor reproductive integrity besides cell death with conventionally fractionated radiation is mainly by DNA damage. However, advanced conformal radiation delivery enables the use of much higher doses of radiation (hypofractionation), such as stereotactic ablative RT, which allow daily doses of radiation (8 to 20 Gy) to be delivered safely. High doses of radiation appear to induce cell death in a manner that is DNA damage independent. It is thought to induce endothelial cell death resulting in vascular damage as well as increased T-cell priming in draining lymphoid tissues [10]. These advanced radiation deliveries are important as it appears that hypo-fractionated radiation may be more effective than conventionally fractionated (2 Gy/d) in inducing an abscopal effect when combined with IT, cytotoxic T-lymphocyte antigen-4 (CTLA-4) inhibition [11-12]. Brachytherapy is commonly used to treat several types of malignancies such as uveal melanomas (UMs) and prostate cancer. However, there are limited data on brachytherapy use in combination with IT, but it may be another means to induce an abscopal response. Some 25% of patients of prostate cancer treated with brachytherapy developed antibodies to tumor-associated antigens [13]. Similarly, UMs treated with eye plaques also demonstrated an increase in immune response, by expression of tumor antibodies [14-15]. Also, few case reports suggest a potential systemic effect related to brachytherapy in patients with choroidal melanoma [16]. Preclinical studies have proposed that brachytherapy may induce a similar increase in Fas expression to induce an abscopal response [17]. The abscopal effect requires priming of immune cells against tumor antigens, like immune reactions [18]. Abscopal effects of RT is enhanced when combined with immunomodulatory drugs like ipilimumab, pembrolizumab, etc., which induces the systemic anti-tumor immune response [19].

**Figure 1:**
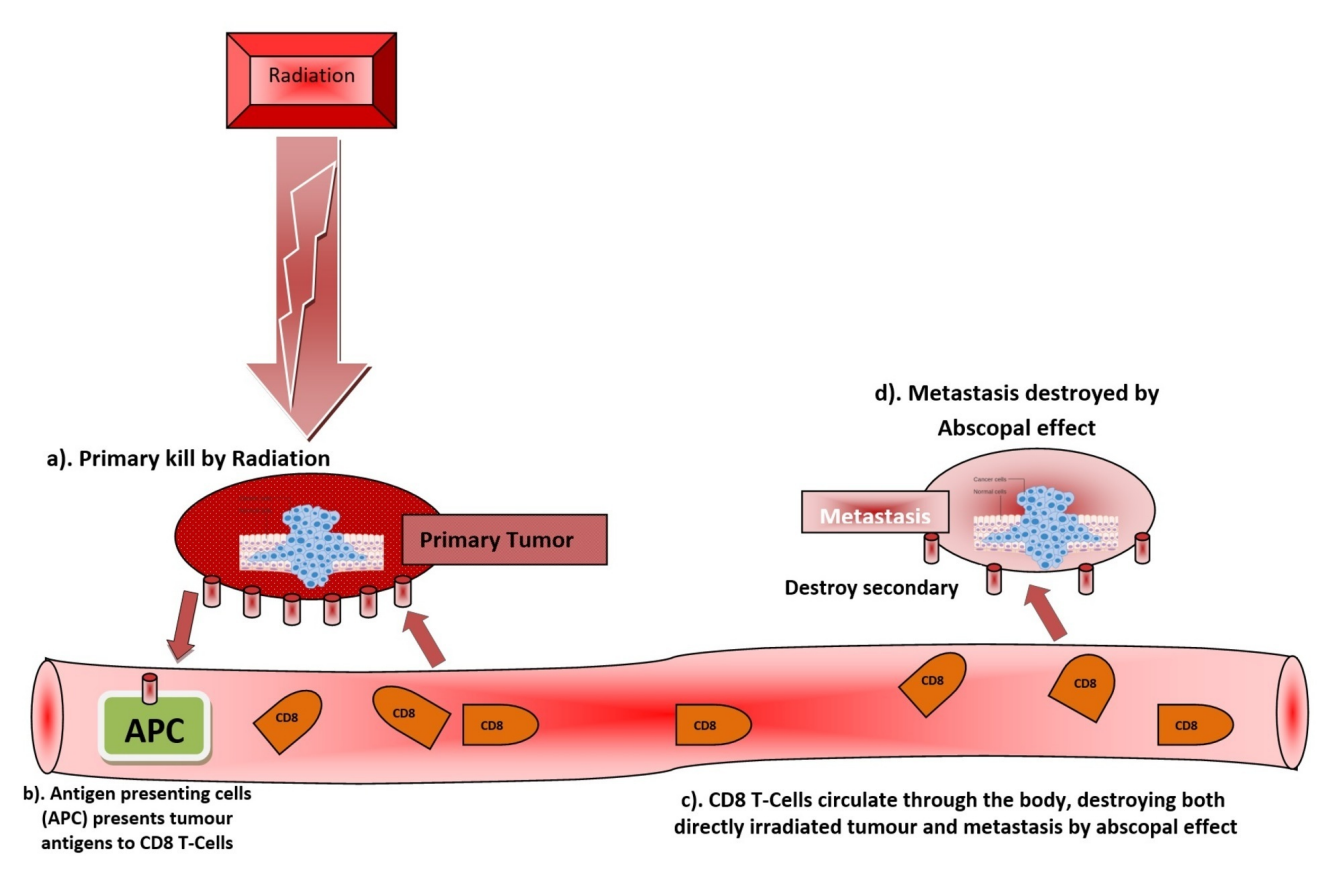
Hypothesis and mechanism of abscopal effect and its immunomodulation (adapted and modified from Wikipedia).

Confined irradiation of a primary tumor leads to tumor cell death coupled with likely possible immunogenic response and liberation of tumor cell-derived antigens, which are recognized and processed by antigen-presenting cells (dendritic cells and macrophages). The cytotoxic T-cells are then primed by recognizing these antigens on and by the tumor antigen-presenting cells and then circulated through the blood stream to destroy the remaining tumor cells in the unirradiated distant parts of the body as shown in Figure 1. Consequently, this increase in tumor-specific cytotoxic T-cells shows a relationship with abscopal anti-tumor responses of RT [2]. Abscopal effects of ionizing radiation are currently under intensive investigation, but so far no consensus on the optimal radiation regimen is needed to increase the efficacy of abscopal tumor regression.

Review in clinical practice

Abscopal effect has been studied extensively in various malignancies. Melanoma is a highly immune-mediated malignancy associated with high infiltrate of immune cells such as melanoma-specific tumor infiltrating lymphocytes having a prognostic value [20]. There had been a considerable effort to harness the immunogenicity of melanoma for therapy. Indeed, this has shown better outcome in comparison to chemotherapy, but the outcome of metastatic melanoma nevertheless remains low.

The first of such immune-regulating agents approved by the FDA to treat patients with metastatic melanoma was ipilimumab, a CTLA-4 inhibitor for clinical use in early 2011, and since then, several reports of abscopal effect have emerged, in order to induce enhanced tumor clearance [2, 21-22]. In a study of 47 consecutive metastatic melanoma patients treated with ipilimumab, RT was analyzed for abscopal response by assessing the response of an index lesion that was outside the radiation treatment field. The study found that in 16 patients (25%), the index lesion decreased in size following radiation out of which 11 index lesions enlarged before radiation. The median survival for their cohort was 28 months. This study also revealed that the abscopal effect appears to be more effective with a moderately hypo-fractionated dosage of less than 3 Gy as compared to a higher dose of hypofractionation of >3 Gy which leads to effective cell kill, besides destroying the cells responsible for immunomodulation hence lessening the abscopal effect. Robust research with different fractionation schedules is required in humans to identify optimal dose required for reduction of index lesion only. It is unclear whether metastatic disease elsewhere in the body was also regressing [22]. It is now apparent that response criteria for cytotoxic agents developed by the World Health Organization (WHO), and later revisited by the Response Evaluation Criteria in Solid Tumors (RECIST) Group, may not accurately capture the disease response to IT [23].

For renal cell carcinoma (RCC), though conventionally radiation-resistant, RT has been used to palliate symptomatic RCC metastases [24]. However, a recent review suggested that hypo-fractionated RT (HFRT) of ≥ 5 Gy in a single or a few fractions results in a different tumor radiobiology leading to increased endothelial cell apoptosis through the release of mitochondrial cytochrome C triggered by acid sphingomyelinase (ASMase) induced ceramide release or by de novo synthesis of ceramide [10]. Therefore, HFRT efficiently destroys tumor microvasculature and is expected to have better results in tumors that are highly dependent on angiogenesis, such as RCC. This is supported by the excellent local tumor control of HFRT [25]. HFRT has been proven to be safe in the treatment of oligometastatic disease. A systematic review by Kothari et al. reported one-year local control rates of 88% and 86% for intra- and extracranial metastases, respectively, and Grade 3–4 toxicity ranged between 0% and 6% [26]. Similarly, a phase II trial for brain metastases from radio-resistant primary tumors, including RCC, showed median survival rates with stereotactic radiosurgery (SRS) comparable to surgical series [27]. Extracranial HFRT in metastatic RCC or inoperable primary RCC showed local control in 98% of treated lesions, in a prospective phase II trial making it an excellent alternative to metastasectomy for treatment of extracranial metastases that are technically inoperable [28]. Future randomized trials necessitate to confirm the added benefit of HFRT over the conventional RT. The encouraging results of HFRT might also be explained by the effect RT has on the immune system [29].

The synergistic immunomodulatory properties of RT and IT are also promising. Interleukin-2 (IL-2) is known to stimulate helper T-cell (Th1) responses and has been observed to induce complete responses when combined with RT in RCC may improve clinical outcome [30]. A phase 1 study evaluating the combination of SBRT and IL-2, could not detect any dose-limiting adverse effects related to SBRT. Synergistic therapy was linked to an increased frequency of proliferating early effector CD4+ memory T-cells in the peripheral blood leading to better immunomodulatory response [31].

The promising outcome of abscopal effect in a breast cancer patient with multiple metastatic sites was reported by Azami et al., where the patient received localized palliative RT to the breast tumor and some of the painful bone metastases at high fracture risk and received no systemic therapy, due to poor performance of Eastern Cooperative Oncology Group (ECOG) status/score. A positron emission tomography (PET) scan for assessment revealed a dramatic disease remission, not only in all the irradiated sites, but also in all the metastatic sites originally exhibiting abnormal fluorodeoxyglucose (FDG) uptake [32].

Dewan et al. applied several RT regimens to assess the effect of different fractionation on the abscopal effect. They injected histone deacetylase inhibitor trichostatin A (TSA) mouse breast carcinoma cells into mice and were randomly assigned to eight groups receiving no RT or three distinct regimens of RT (20 Gy × 1, 8 Gy × 3, or 6 Gy × 5 fractions in consecutive days) with or without specific monoclonal antibody (9H10), against cytotoxic T-lymphocyte associated protein-4 (CTLA-4). The results showed that abscopal effect occurred only in mice treated with the combination of 9H10 and fractionated RT (p < 0.01). Fractionated but not single-dose RT induced an abscopal effect in this study [11].

The abscopal effect in a hematological malignancy was first reported by Li in 1963 [33] and described the leucocytopenic effect of splenic irradiation in patients with leukemia. Similar phenomenon was described by Nobler [34] in 1969 in a patient with lymphoma and subsequently multiple case reports followed thereafter in lymphoma patients [35-36]. The suggested mechanism of the abscopal effect in lymphoma is thought to be largely due to cancerous lymphocyte damage circulating through the spleen during irradiation and results in the overall regression of malignancy [37]. This is different from the postulated mechanism in solid malignancies, which is thought to be due to the release of various cytokines and inflammatory markers such as tumor necrosis factor (TNF) [38]. Other aspects of the immune system have been implicated as well in the abscopal effect, for example, the enhanced response of natural killer cells in some cases [39].

## Conclusions

The abscopal effects of RT have been extensively reported in preclinical and clinical studies whereby irradiated tumor cell death can stimulate anti-tumor adaptive immunity by promoting the release of tumor antigens and their cross-presentation to T-cells. In order to overcome the immune-resistance of malignant tumors to RT alone, cancer IT especially immune checkpoint inhibitors, the abscopal effect of RT has become more meaningful by the synergistic approach of RT and IT combination. The increasing use of high dose per-fraction RT approaches also offers the possibility that novel combinations with current systemic strategies could enhance systemic anti-tumor effects. A meticulous approach is required to incorporate the current understanding of abscopal signaling with the aim of developing novel systemic agents to control tumor expansion, besides also defending normal tissues when combined with RT.
